# Multi-institutional MRI-based radiomic pilot study to measure the variations between scanner vendors and imaging sessions

**DOI:** 10.3389/fonc.2026.1686601

**Published:** 2026-03-03

**Authors:** Suong Duong, Danny Lee, Carri Glide-Hurst, Bhudatt Paliwal, Jennie Crosby, Michael Boss, Yunfeng Cui, Mi Huang, Heng Li, Khadija Sheikh, Taeho Kim, James Monroe, Jung Hun Oh, Ying Xiao, Jason W. Sohn

**Affiliations:** 1 Department of Environmental Protection, Pennsylvania Commonwealth, Pittsburgh, PA, United States; 2 Department of Radiation Oncology, Allegheny Health Network, Pittsburgh, PA, United States; 3 Department of Human Oncology, University of Wisconsin-Madison, Madison, WI, United States; 4 Center for Research and Innovation, American College of Radiology, Philadelphia, PA, United States; 5 Radiation Oncology, Duke University Hospital, Durham, NC, United States; 6 Department of Radiation Oncology, Medical College of Wisconsin, Milwaukee, WI, United States; 7 Department of Radiation Oncology, Johns Hopkins University, Baltimore, MD, United States; 8 Department of Radiation Oncology, Washington University in St Louis, St. Louis, MO, United States; 9 Department of Radiation Oncology, St Louis VA Medical Center-Jefferson Barracks, St. Louis, MO, United States; 10 Department of Medical Physics, Memorial Sloan Kettering Cancer Center, New York, NY, United States; 11 Department of Radiation Oncology, University of Pennsylvania, Philadelphia, PA, United States; 12 Department of Radiation Oncology, Beth Israel Deaconess Medical Center, Harvard Medical School, Boston, MA, United States

**Keywords:** 3D printing, MRI, multi-institution, phantom, radiomic features

## Abstract

**Background:**

Multi-institutional clinical trials frequently use MRI imaging for critical decisions and guidance for medical treatments. Collecting and analyzing images produced by various MR vendors and models is quite difficult since image quality can be highly variable. No unifying quality control targeting protocol studies exists to ensure MRI images used in that study are comparable. This project will investigate variations between imaging sessions and between various scanners using radiomic parameters from prototype MRI QA phantom.

**Purpose:**

To develop a 3D radiomic phantom for quantifying radiomic feature consistency between MRI scanners across multi-institutions.

**Methods:**

The prototype phantom consists of five 3D-printed objects (3 grid and 2 egg-shape) using Polylactic Acid (PLA) with/without 20% wood particles placed in a water container. The grid objects consisted of PLA scaffolding with 245 cubic voids (flood-filled by water) stacked in 7rows x 7columns x 5layers with volumes of 3x3x3 mm^3^ or 5x5x5 mm^3^, and scaffolding thickness of 1mm or 2mm. The egg-shaped objects are 5cm long with a 2cm or 4cm maximum diameter, filled with vitamin D3-capsules and olive-oil. It was scanned 10 times using T1- and T2-weighted sequences on Philips (1.5T Elekta Unity), GE (1.5T, Signa Artist), Siemens (1.5T MAGNETOM Sola), and Philips (1.5T Ingenia) across four institutions. TrueFISP and T2w sequences were used on ViewRay (0.35T MRIdian) scanners at two institutions. Per object, 107 radiomic features were extracted using the Pyradiomics extension in 3D Slicer. Coefficients of Variation (CV) of individual radiomic features were compared across 10 scans acquired on each scanner and used to compare radiomic feature consistency between objects and MRI scanners.

**Results:**

The radiomic feature consistency varied across objects with less reproducibility for the egg-shaped objects and more reproducibility for the grid objects, with slightly better reproducibility for T1w than T2w sequences. The GE scanner demonstrated better reproducibility than the other scanners. Both ViewRay scanners showed consistency for acquisitions with the TrueFISP sequence; the median CV of 107 radiomic features between objects was <10%). The consistency was summarized in a heat map.

**Conclusion:**

Some radiomic features showed significant intra-scanner variations. This study demonstrated that a standardized radiomic phantom is required to characterize individual scanners and MR sequences for establishing the baseline of radiomic features, which could be important for multi-institutional radiomic studies using MRI.

## Introduction

### Radiomics – definition, features, and application

Radiomics is a field of medical research that involves the use of advanced computer algorithms to extract a large number of quantitative features from medical images, such as computed tomography (CT) (1), Cone Beam CT (CBCT) (2, 3), positron emission tomography (PET) (4) and magnetic resonance imaging (MRI) scans (5) for mineable data. There are many different types of radiomics features that can be extracted, including shape features (e.g., volume, surface area), voxel-based intensity features (e.g., mean, standard deviation), and texture features (e.g., entropy, homogeneity) (6, 7). These quantitative features can be used to characterize and classify the appearance and structure of tissues and organs within the body (7). Specifically, radiomic analysis extracts quantitative features from images to uncover characteristics unable to be identified visually by a human observer (8).

Radiomics has been applied to a wide range of medical specialties, including oncology (9), neurology (10), and cardiology (11), and has the potential to improve diagnosis, prognosis, and treatment planning. In oncology, radiomics features can be used to stage individual patients, predict patient outcomes, stratify patients into subgroups, and monitor disease progression (12).

More specifically, many studies have demonstrated the potential of MRI radiomic features for their prognostic and predictive capabilities in cancer, including predicting progression-free survival in locally advanced rectal cancer (13), cervical cancer (14), nasopharyngeal carcinoma (15), and breast cancer (16). A comprehensive literature review assessed the use of MRI radiomics in brain tumor classification, highlighting its potential as a valuable tool for glioma grading and differential diagnosis (17). Radiomics features have been used to predict the aggressiveness of tumors and to identify patients who are more likely to respond to certain treatments (18).

### Reproducibility assessment

Radiomics is subject to certain limitations and sources of variability that can affect its robustness and reproducibility (19, 20). Some of the factors that can impact the effectiveness of radiomics include the quality of the imaging data, the selection of the features that are extracted, and the methods that are used to analyze the data.

In addition, radiomics studies should be designed and conducted in a manner that promotes reproducibility. This can include using standardized protocols for image acquisition and analysis, clearly documenting the methods and analysis steps that were used, and making the data and code available for other researchers to review and reproduce the results. By following these best practices, researchers can help to ensure the robustness and reproducibility of their radiomics findings.

Radiation treatments for patients are designed by simulating the distribution of radiation dose on 3D models of the patient using computer modeling (‘treatment planning’). While these models are traditionally created using CT images of the patient, MRI plays an increasingly important role in radiotherapy due to its superior visualization of soft tissues. Radiomic features unique to MR images can provide additional information that improves treatment outcome modeling.

Previous publications have established that reducing the noise (or uncertainties) within the quantitative data could improve the prediction accuracy and move this type of work closer to clinical practice. A multi-institutional study investigated quantitative metrics for Dynamic contrast-enhanced (DCE)-MRI and found that DCE-MRI performed relatively stable in digital reference objects (DROs) with relatively low noise. However, in patient data with lower signal-to-noise ratio (SNR) and different acquisition parameters, quantitative evaluation became challenging. The authors therefore recommended to standardize acquisition and analysis parameters for DCE-MRI, especially in multi-institutional clinical trials (21, 22).

Another study evaluated image variabilities for 100 CT scanners across 35 clinics by imaging a radiomics phantom using a control protocol and a local protocol (23). By using the control protocol, the overall variability was reduced by >50% compared to the local protocol. These studies demonstrated the importance of understanding inter-scanner variability, standardizing data acquisition protocols, and establishing reference baselines for radiomic studies.

### Previous phantom approaches

Researchers have repurposed existing phantoms for use in MRI radiomics studies (24–26) or customized them using inserts consisting of polystyrene beads and spheres in agar gel (27, 28). Lee et al. (29) built an MRI radiomics phantom using 20 cylinders of material with varying radiomics feature properties and tested its utility across varied protocols (varied number of excitations, slice thickness, phasing steps, and field of view) on 2 MRI scanners (Siemens and Philips 1.5T). Scans were performed twice to evaluate reproducibility. According to their results, most intensity-based and GLCM features had an intermediate (10% ≤ CV < 30%) or small (CV < 10%) CV, whereas most NGTD features had a high CV (CV ≥ 30%).

Bush et al. (30) used a publicly available MRI dataset that were acquired by three different MR scanners on a phantom that was comprised of eighteen 25‐mm doped gel‐filled tubes and a single 20‐mm tube containing 0.25 mM GdDTPA. They reported significant differences in many texture features calculated from these MR images, which is consistent with the findings from our study. Mayerhoefer et al (31) used two custom-made phantoms consisting of polyethylene tubes filled with polystyrene spheres and an agar gel solution to study the impact of different MR scanning protocols on the radiomics analysis. In their study, texture features from all categories were found to be sensitive to the different imaging protocols, without evidence that any particular category of features performed worse. Waugh et al (32) used a breast-mimicking phantom that had four different samples of reticulated foam submerged in tubes filled with agarose solution. The purpose of using this phantom in their study was to measure the ability of radiomics features in distinguishing different texture objects under different imaging conditions.

Xue et al. studied (33) the repeatability and reproducibility of radiomics features on prostate cancer patients who were imaged using a 1.5T MR simulator (Ingenia MR-RT, Philips Healthcare) that was equipped in a MR-LINAC (Elekta Unity, Elekta). Each patient had two MR scans on the same day and a third scan within 7 days using a standardized 3D T2w turbo-spin-echo (3D-T2W-TSE) sequence. Only a small amount out of 1023 radiomics features investigated in their study had good repeatability and reproducibility, indicating the importance of feature robustness study for this type of MR scan and anatomical site. It should be noted that the repeat imaging study involving real patients is subject to variation in patient anatomical and physiological changes.

The low-field MR-Linac showed a high degree of variability as compared to the other MRI scans evaluated although limited phantom data are available for comparison on this system. Ericsson-Szecsenyi et al. (26) performed repeated radiomics measures of their clinical TrueFISP sequence on two commercially available phantoms not designed for radiomics endpoints and nevertheless, found that the vast majority of features extracted were robust (defined as CV < 5%, (Coefficient of variance)). More prevalent in the literature is delta-radiomics (e.g., temporal characterization of images acquired before, during, and after treatment) as a means to quantify tumor and normal tissue response assessment. Cusumano et al. (34) led a multi-institutional external validation study in 43 locally advanced rectal cancer patients imaged longitudinally across 3 low-field MR-Linac programs; results were mixed and they concluded that other image-based biomarkers such as early-regression index were more promising.

Fruit and vegetable arrays have served as phantoms, providing diversity of textures and shapes, but suffering from their ultimately ephemeral nature (35–37). Diverse materials-focused phantoms informed by feature values seen in patients’ imaging have been used to assess reproducibility and robustness across time and scanners (29). Recent efforts to develop geometrically complex phantoms have utilized 3D-printing to evaluate the reproducibility of radiomic features across sites, demonstrating the importance of standardized test objects and acquisition protocols (38). MR images generated from virtual phantoms have also been used to generate radiomic feature sets (39–41).

### Need for improved standardized reference objects

Having consistent reference objects facilitates direct comparisons of scanner technical performance when generating radiomic features. While previous efforts to assess scanner technical performance in radiomics have demonstrated the robustness of certain features, the reliance on fruits and vegetables provides less confidence in the reproducibility of results, as the material properties responsible for differentiation of radiomic feature values can vary from specimen to specimen. It is difficult to control the structure and length scale of physical features and material properties in fruit, tempering their utility for cross-site and longitudinal assessment of radiomic feature reproducibility. While digital reference objects are stable, they are not always representative of the clinical imaging experiment and are unable to assess the technical performance of scanner hardware, being limited to testing only the reproducibility of software tools and pipelines involved in radiomic feature set generation. For this reason, a well-designed, stable physical phantom with known geometry and material composition is desirable to assess the technical performance of scanners when generating radiomics feature sets from medical images. We designed five objects that could test wide range of radiomic parameters. We planned to investigate three perspectives; firstly, how much radiomic parameters can be consistent between institutions, secondly, how much they vary between imaging sessions within an institution, and thirdly, the extent to which radiomic parameters vary depends on the material properties and shapes of the objects. In our study, the robustness and reproducibility of MRI images, with a novel phantom development, has been evaluated at scanners across multiple institutions. Shape, statistical, and texture features including GLCM (Gray Level Co-occurrence Matrix), GLDM (Gray Level Dependence Matrix) GLRLM (Gray Level Run Length Matrix), GLSZM (Gray Level Size Zone Matrix), and NGTDM (Neighboring Gray Tone Difference Matrix) were extracted from the images acquired on MRI scanners at multiple institutions (42).

## Methods and materials

### 3D phantom design

**Figure 1:**
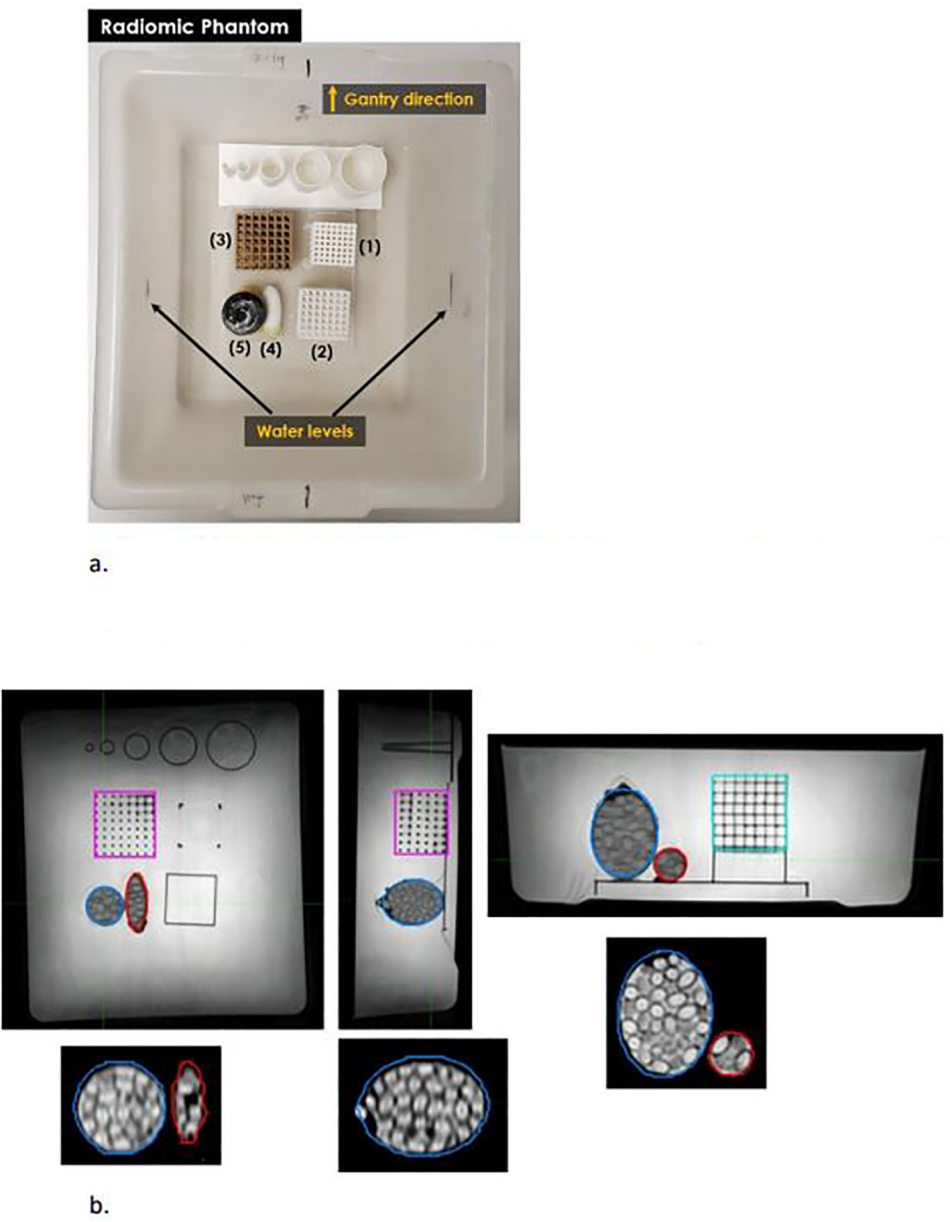
**(a)** Image of the 3D printed objects and container comprising the radiomics phantom. (1)Grid 3x3x2, (2) Grid 5x5x1, (3), Grid 5x5x2, (4) Egg 5x2, and (5) Egg 5x4. **(b)** T2w image of the radiomics phantom from the 1.5T Philips Unity scanner.

We designed, 3D-printed, and assembled an MRI-safe phantom consisting of five objects with varied sizes, textures, and shapes (three grid and two egg-shaped, **Figure 1a**) using AutoCAD (AutoCAD 14, Autodesk, USA) and ideaMaker (Raise 3D Technologies, Inc, Irvine, CA, USA). We printed the three grid objects with a PRUSA i3 printer (PRUSA Research, Czech Republic) and the egg-shaped objects with a Raise3D Pro2 printer (Raise 3D Technologies, Inc, Irvine, CA, USA). We used two materials to print: Polylactic Acid (PLA) with 20% wood particles (for the 5 × 5 × 5 mm^3^ brown grid with a 2 mm thickness) and PLA without wood particles for the other four objects. Both PLA filaments had a 1.75 mm diameter with +/- 0.03 mm dimensional accuracy. Printing temperatures were 180 °C to210 °C.

Shown in **Figure 1a**, Grid 3x3x2 consists of a 7×7×5 mesh object with 3×3×3 mm^3^ interiors formed by 2 mm thick framing. **Figure 1b** shows the objects in T2w. This forms a frame 30 mm wide, 30 mm long, and 25 mm high. Grid 5x5x1 and Grid 5x5x2 consisted of 5×5×5mm^3^ interiors formed by 1 mm and 2 mm thick framing, respectively. The frames were stacked into 7×7×5 mesh forming objects 42x42x30 mm^3^and 49x49x35 mm^3^. The two hollow egg-shaped objects are: 1) Egg 5x2 (5 cm long, 2 cm maximum diameter) formed by a 1 mm thick shell and 2) Egg 5x4 (5 cm long, 4 cm maximum diameter) formed by a 1 mm thick shell. Both egg-shaped objects were packed with vitamin D3 capsules (125 mg, 5000 IU, Nature Wise brand) to simulate tissue and filled with olive oil to eliminate air between capsules. Olive oil was used instead of water to prevent the capsules from dissolving. All five objects were fixed in position within the container. The phantom is filled with distilled water for imaging, minimizing air bubbles. The detailed dimensions of the objects are noted in the drawings in Appendix Figure 1.

### Stability of the egg-shaped objects’ contents

To evaluate possible degradation of our tissue substitute, Vitamin D, we stored a test group of 480 capsules in olive oil for 26 weeks and then compared to a control group. The capsules in the control group were kept dry in bottles. Ten capsules from the test group were randomly selected per 2-week and cleaned with 100% pure acetone three times to remove oil. After air drying for 30 minutes to remove acetone, the capsules’ diameter, weight, and shell thickness were measured and compared to the capsules in the control group (480 capsules).

### MR imaging for multi-institutional radiomics evaluation

To provide benchmarking data across different end-users and MRI scanners, data collection was performed at 4 different institutions.

#### Institution A

Institution A utilized an Elekta Unity, which is constructed with a Philips 1.5T scanner (Elekta Unity MR-Linac, Elekta; Stockholm, Sweden). Ten MR scans were performed with three different time intervals on the Unity with two MR receiver coil systems: a 4-channel anterior coil, and a 4-channel posterior coil. The scan sessions were performed in three sets: (1) one-week intervals between the first 3 scans, (2) one-day intervals for the next 3 scans, and (3) a minimal interval (i.e., re-setup the radiomic phantom) for the last 4 scans within 24 hours. During each MR imaging session, 3D T1w and T2w sequences were acquired. All sequence parameters are summarized in Appendix Table 1.

#### Institution B

Institution B has a ViewRay (MRIdian) and GE 1.5T (Artist MR-SIM). The test phantom was scanned 10 times using T1w and T2w sequences for both machines. In addition, scans using the TrueFISP sequence were acquired on the ViewRay. Since the gantry angle can affect image distortions and quality, we maintained a consistent gantry angle during the measurements. The system’s home angle is set at 300 degrees, as this angle provides the best image quality for clinical delivery in terms of distortion and MRI isocenter shift. Therefore, we conducted the measurements at the home angle of 300 degrees.

#### Institution C

Institution C used a Siemens 1.5T scanner (Magnetom Sola) and scanned the phantom 10 times; T1w and T2w TSE axial scans were acquired.

#### Institution D

Institution D scanned the phantom using a ViewRay TrueFISP sequence and T1w and T2w sequences on a Philips 1.5T MR Ingenia scanner.

**Appendix Table 1** summarizes the MRI sequence parameters for each scanner. As shown in the table, T1 and T2 sequence parameters are different between various vendor’s scanners.

### Radiomic parameter calculations using pyradiomics

#### Contouring objects and radiomic feature extraction

All scans were imported into an image management system (MiM, Cleveland OH) for manual contouring of the objects (see **Figure 1**). Each MR series and associated contours were then imported into 3D Slicer (3D Slicer, Version 4.11.2) for contour-based feature extraction using the Pyradiomics extension (42). The settings used for extracting features were: 64 fixed bin width and enforced symmetrical GLCM (gray level co-occurrence matrix). The 107 extracted radiomic feature values for the 5 objects were saved as a comma-separated value text file for further analysis. We used one segmentation set by performing image registrations between all image sets.

#### Feature analysis

##### Coefficient of variation

Using coefficients of variation (CV (%)), we analyzed the 10 values of each radiomic feature from the 10 repeated MR image sets to investigate intra- and inter-scanner variabilities. Coefficients of variation for a radiomic feature in an object was calculated by (29).


$$CV\;(\%)=\;\frac{std}{mean}*100\%$$

in which, std and mean were standard deviation and mean of the feature value over the 10 repeated MRI scans. CVs were classified into three levels: <10% was considered high consistency (small variation), 10 to 30% was considered moderate consistency (moderate variation), and >30% was considered weak consistency (high variation) (29).

For the intra-scanner variability analysis, CVs were calculated between objects and sequences in (a) 1.5 T Philips Unity at Institution A, (b) 1.5T GE MR simulator and 0.35T ViewRay at Institution B, (c) 1.5T Siemens MR simulator at Institution C, and (d) 1.5T Philips Ingenia MR simulator and a ViewRay at Institution D. We analyzed intra-scanner variability and inter-scanner variability from multiple image sets using the same phantom.

##### Intraclass correlation coefficients

Intraclass correlation coefficients were used to evaluate the variation between the same two vendor and model scanners (ViewRay 1 vs ViewRay 2) for the same phantom objects and shown in **Appendix Figure 2**. The ICC(3,1) method was used and calculated by (43) using the equation below,


$$ICC(3,1)=\;\frac{MS_{o}-MS_{E}}{MS_{o}+(k-1)*MS_{E}}$$

In which $\;MS_{o},\;MS_{E}$: are the mean square of objects (value of each object was the average value of that object over 10 scans) and mean square of error (mean square deviation). k is the number of scanners. In general, three levels of ICC including < 0.75, 0.75 to 0.9 and > 0.9 were considered as bad, good, and very good, respectively.

Absolute values of ICC were used to determine which radiomic features had strong correlations between the two scanners.

#### Software development for data analysis using Python

Feature analysis was performed with Python 3 (version 3.9), using the matplotlib library for plotting. PyQt5 was used to build the GUI interface for the software using Python 3 scripts. The software was designed to process multiple radiomic datasets including feature extractions using Pyradiomics and results were stored in csv format. The software produces and displays analysis tables and plots of all 107 radiomic features.

## Results

### Phantom material consistency over time

Vitamin D3 capsule weight and diameter did not change appreciably over time. The control (dry) and test (immersed in olive oil) groups’ diameter mean over 26 weeks were 6.31 mm and 6.50 mm with standard deviations of 0.07 mm and 0.04 mm, respectively. The mean weights of control and test groups were 0.23 g and 0.24 g, with standard deviations 0.002 g and 0.001 g, respectively. No appreciable changes demonstrating capsule degradation were observed over 12 weeks.

### Intra-scanner variation comparisons of five scanners

**Figure 2:**
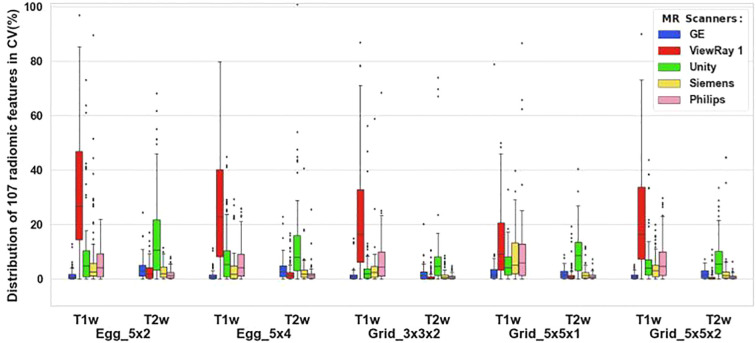
Coefficients of variation for each radiomics test object for T1w and T2w sequences acquired on five scanners. Coefficients of variation for each radiomics test object are processed as a combination of variables for T1w and T2w sequences acquired on five scanners. The coefficients of variation of each object of the 107 radiomic features were calculated and plotted using boxplot.

We calculated radiomic features from 10 scans acquired on each scanner and processed the data for each phantom object. The boxplots in **Figure 2** best summarize the CV per each test object for T1w and T2w sequences across all five scanners and 107 radiomic features.

**Figure 3:**
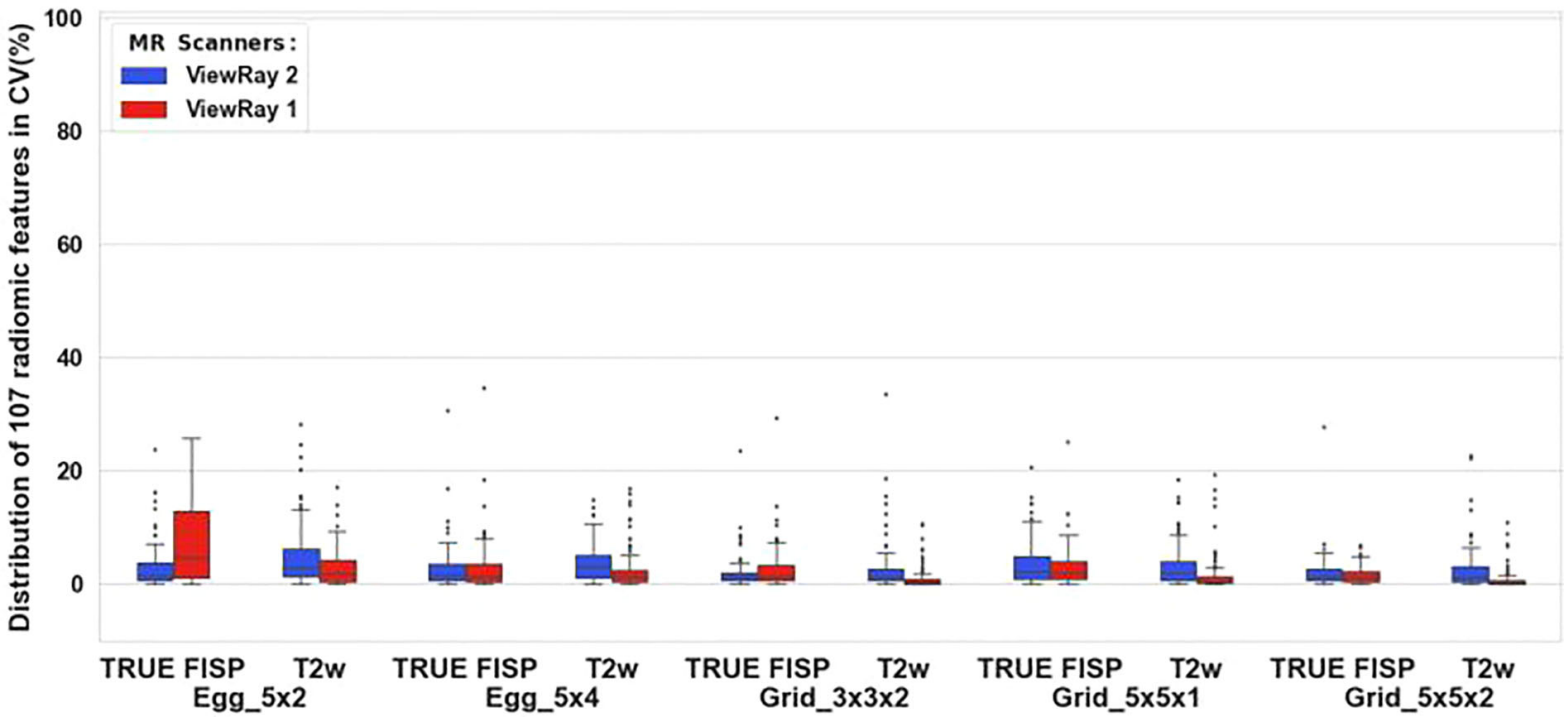
Radiomic feature consistency of 5 objects across 2 sequences of the 0.35 T ViewRay scanner. The consistency is shown by the distribution of the coefficients of variation CV (%) in all radiomic features of each object and each sequence.

T1-weighted imaging on ViewRay showed the largest variation for all five objects in **Figure 2**; however, TrueFISP (TRUFI) is the sequence most commonly used in clinical ViewRay workflows. Two institutions submitted TrueFISP images sets for comparison between two Viewrays as shown in **Figure 3**. The CV for the two sequences were generally low, mostly below 10% except the TrueFISP sequence for the Egg_5x2 object.

### Inter-scanner variability of the 7 radiomic feature groups

**Figure 4:**
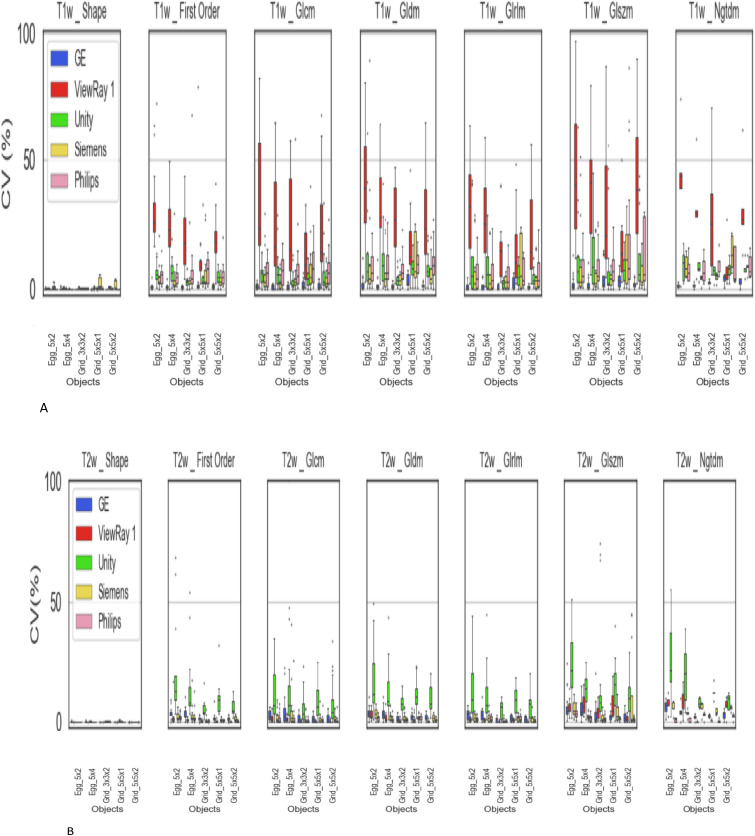
**(A)** Radiomic feature variation for the 7 radiomic groups. CV variations between 7 groups in ten T1w. **(B)** Radiomic feature variation for the 7 radiomic groups. CV variations between 7 groups in ten T2w.

Pyradiomics organizes radiomics features into seven groups (described in Appendix Table 2). We processed the radiomic data for each group and compared the intra-scanner (ten imaging sessions) variations between scanners. For example, in **Figures 4A**, within the First Order Statistics group (the second plot), there are 18 features. For each feature, we calculated a coefficient of variation (CV) from 10 repeated scans. This resulted in 18 CV values per scanner, which were summarized as a box plot for each scanner. We repeated this process for five scanners to visualize and compare inter-scanner variability. Smaller variability between scanning sessions indicates better repeatability, and the variation of First Order features was compared across scanners. For T1-weighted images, the GE scanner showed the least variation. For T2-weighted images, all scanners exhibited lower variation compared to T1-weighted images, while the Unity scanner showed the largest variation among them. Shape-based radiomic features were very consistent and less than 7% variation was observed for both T1w and T2w as shown in first column of graphs in **Figures 4A, B**.

Between phantom objects modeling tumors (grid vs. egg-shaped types), there were some differences for both T1w and T2w image sets as shown in **Figures 4A, B**, 2^nd^ column to the 7^th^ column. Each object’s CV varies between T1w and T2w per radiomic group. This may be due to the characteristics of object types (grid vs. egg-shaped). The ViewRay scanners showed the largest CVs between 10 image sets (intra-scanner variation) for the T1w sequences, however, the T1w sequence is not used clinically.

### Intra-scanner variability per individual features for all objects

**Figure 5:**
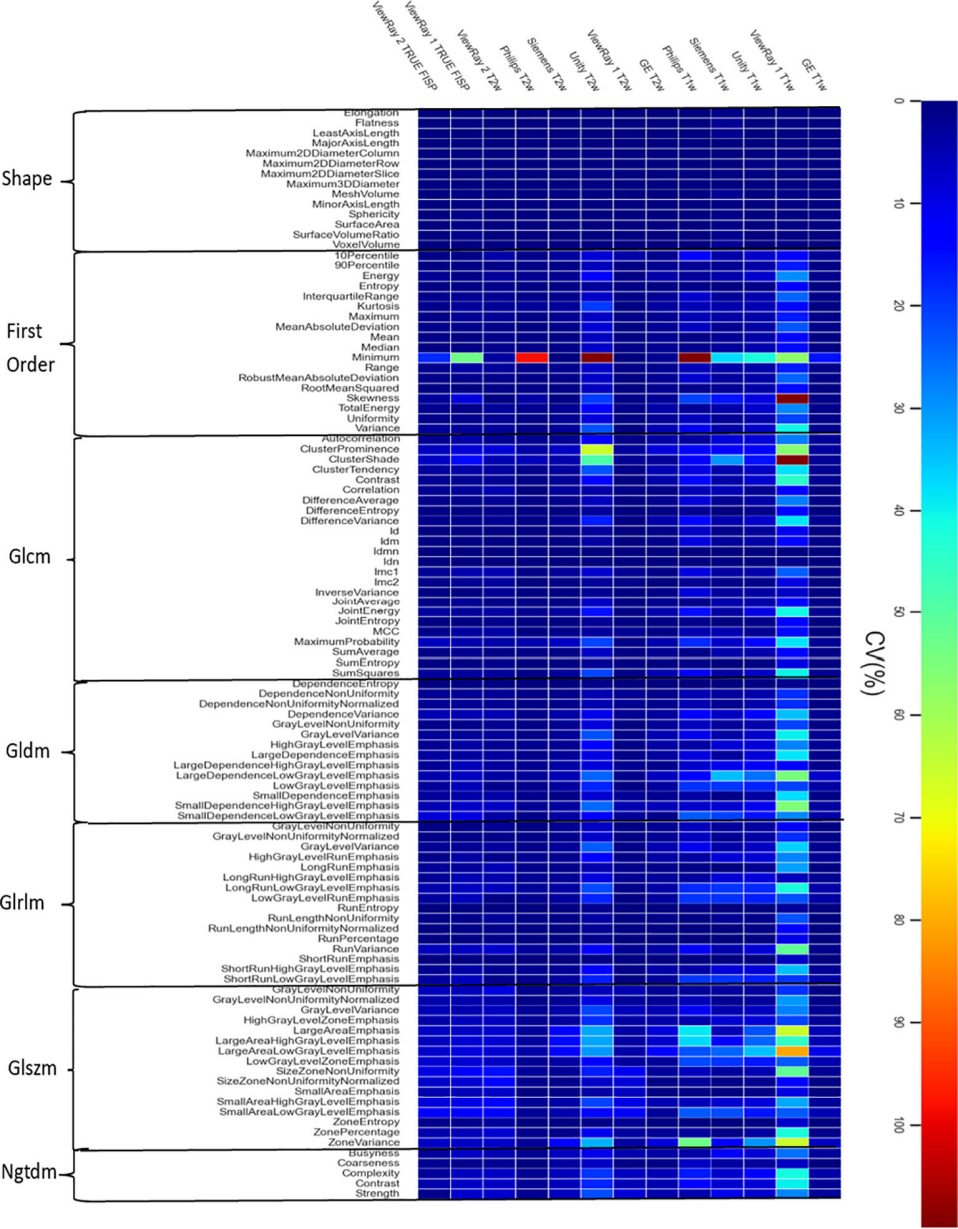
Individual radiomics feature variations display in a heatmap. The detail description of features is in **Appendix Table 2**.

We calculated the CV per radiomic feature, which was displayed in a heat map (**Figure 5**). This map depicts the variations across 10 repeated scans for each radiomic feature. Some of the features were well below 10% CV, however, some demonstrated a higher variability. For example, the Philips Ingenia T1 sequence image sets showed over 40% CV in 4 features.

**Figure 6:**
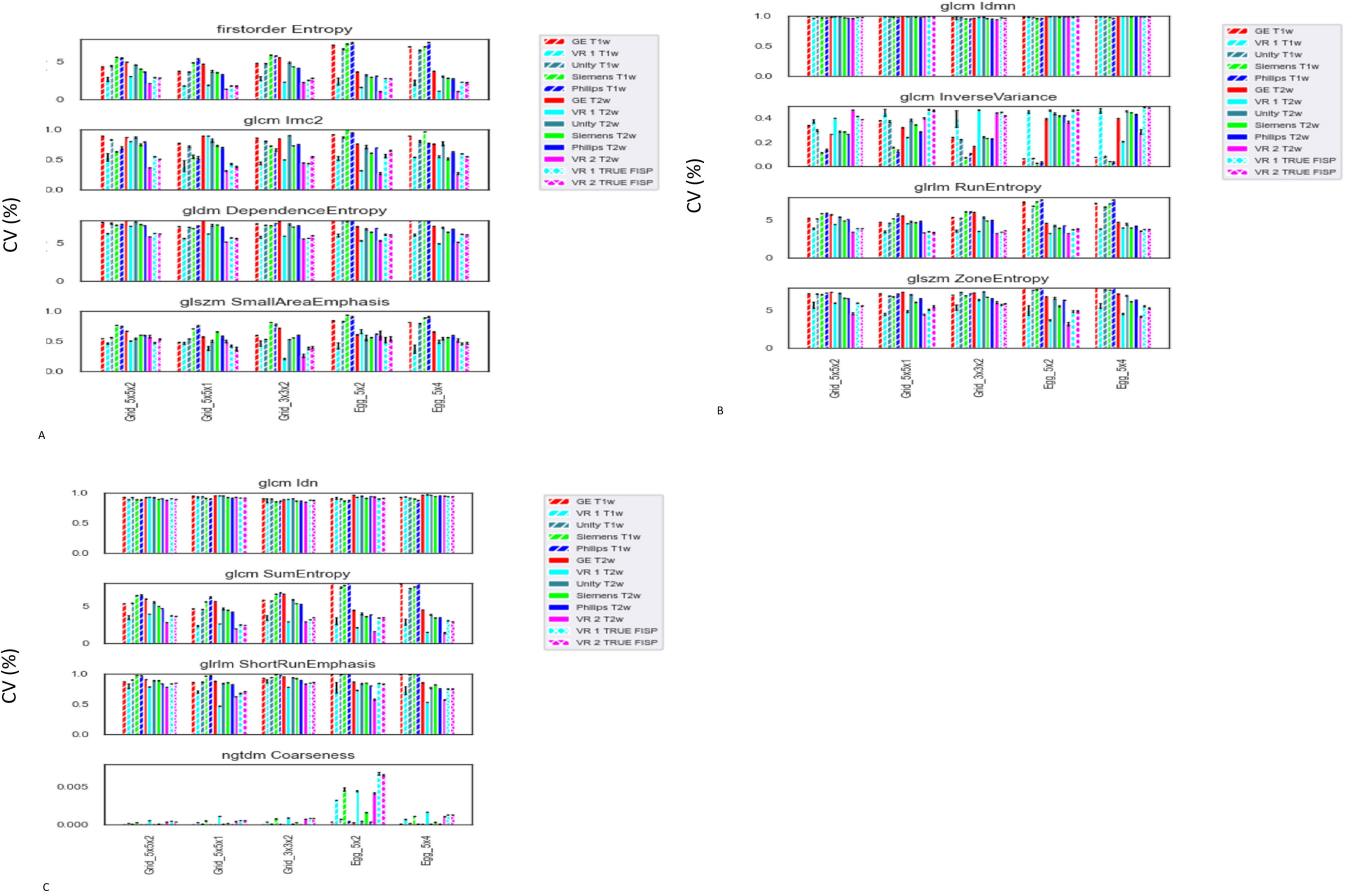
**(A)** Selected plot of raw radiomic feature values from each scanner. One feature per radiomic feature group which showed below 10% CV. **(B)** Selected plot of raw radiomic feature values from each scanner. One feature per radiomic feature group which showed below 10% CV. **(C)** Selected plot of raw radiomic feature values from each scanner. One feature per radiomic feature group which showed below 10% CV.

We selected one feature per radiomic feature group (total of 6 groups: First Order, Glcm, Gldm, GLrlm, Glszm, and Ngtdm except the physical shape group) which showed below 10% CV and compared raw radiomic feature values from each scanner. The results are summarized in **Figures 6A–C**. Even with consistency between scan sessions, actual radiomic feature values were significantly varied except two features (Glcm Idmn and Glcm Idn).

In this research, ViewRay 1 and ViewRay 2 used the same MRI sequence parameters. We wanted to see which radiomic features, and which sequence had the strongest correlation between the two scanners. For the intraclass correlation coefficients, the TrueFISP sequence had the largest number of radiomic features (80/107 above 90%, 95/107 above 75%) demonstrate a strong correlation compared to the T2 sequence (22/107 above 90%, 54 above 75%). In the TrueFISP sequence, three feature groups (shape, first order, and ngtdm) had a higher percentage of radiomic features above 90% than other radiomic groups. In the T2 sequence, only the shape feature group had more radiomic features (>90%) with a strong correlation.

## Discussion

Several prior studies have investigated MRI radiomics reproducibility using phantom-based approaches, providing important benchmarks for scanner, acquisition, and processing variability. Recent work by Cheong et al. (44) demonstrated improved computational and imaging reproducibility of multiparametric MRI radiomics features using a dedicated phantom, with validation extending to clinical brain tumor data. Similarly, Hajianfar et al. (45) systematically evaluated the impact of scanner differences, acquisition parameters, and harmonization techniques on feature reproducibility using a standardized phantom design. Veres et al. (46) compared 3D-printed phantoms with biological phantoms and highlighted material-dependent differences in feature robustness, while Yu et al. (47) examined longitudinal repeatability of MR radiomics features in MR-guided accelerator imaging using phantom measurements.

In contrast to these studies, the current work focuses on a simple, biologically inspired phantom designed to represent fat- and soft-tissue–like characteristics with patterns while enabling repeated scanning across multiple clinical MRI scanners under routine imaging conditions. Rather than emphasizing harmonization or post-processing strategies, our study isolates intrinsic scanner- and sequence-dependent variability by using repeated acquisitions and direct calculation of repeatability metrics. This design allows a transparent assessment of session-to-session variability and inter-scanner differences while maintaining experimental feasibility. The results complement prior phantom studies by demonstrating that even with a minimalistic and easily reproducible phantom, meaningful differences in radiomic feature stability can be detected across scanners and sequences, supporting its potential utility for routine quality assurance and multicenter reproducibility assessments.

We fully packed vitamin D capsules into egg shape shells to minimize the possible shift. Two image samples from image sets taken at the beginning and at the end were visually compared. There was no noticeable shift over time. One phantom was built for this entire study to eliminate any variation between multiple phantoms.

We demonstrated that inter- and intra-scanner image variation significantly influenced some of the radiomic features. Our results suggest that we may need to further investigate the variations within a scanner model and/or vendor and include more institutions to create benchmark radiomic data for multi-institutional radiomic studies.

To facilitate multi-institutional clinical trials with radiomics endpoints and aid clinical decisions, it is imperative that appropriate benchmarking is performed. Our analysis had similar findings as reported by Lee et al. (26); most of the intensity-based (First Order, in our grouping) and GLCM features showed CV < 30% for T1w (other than ViewRay) and T2w images. However, for T1w images, the NGTD CV is intermediate (other than for ViewRay). For T2w images, the NGTD CV is high for the Philips Unity (Philips 1.5T).

It is important to note that our shape-based radiomic features have exceptionally low variability. This is due to the methods used for this analysis, namely that contour sizes and shapes were kept constant over all the images. Therefore, the image mask shapes did not vary between images across all scanners.

We did not have many scanners that were from the same vendor and model. However, we had two ViewRay scanners and investigated how much they were correlated in terms of radiomic features calculated using their MR images. The TrueFISP sequence had more radiomic features (80/107 above 90%, 95/107 above 75%) with very strong correlations compared to the T2 sequence (22/107 above 90%, 54 above 75%). For future research, we plan to include many scanners and investigate the consistency of their acquired images by correlating radiomic features. We may be able to identify radiomic features that are reliable so that those can be used for building clinical outcome models using a multi-institutional study database. PyRadiomics is used for this study, and mostly IBSI (image biomarker standardization initiative) compliant for the most part. PyRadiomics development also includes the standardization effort by the IBSI team. However, there are some differences between PyRadiomics and feature extraction as defined in the IBSI documents. We expect this standardization process is complete in near future.

## Data Availability

Requests to access the datasets should be directed to jsohn3@bidmc.harvard.edu.
