# Supplementary material for: Multi-institutional MRI-based radiomic pilot study to measure the variations between scanner vendors and imaging sessions
Source: Front Oncol. 2026 Mar 3;16:1686601. doi: 10.3389/fonc.2026.1686601 (PMC12992046; doi:10.3389/fonc.2026.1686601)

Appendix

Section A: Radiomic objects' designs and dimensions

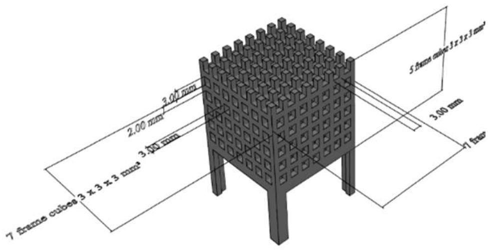

(1) Grid 3x3x2: frame cube 3x3x3 mm^3, wall thickness 2 mm

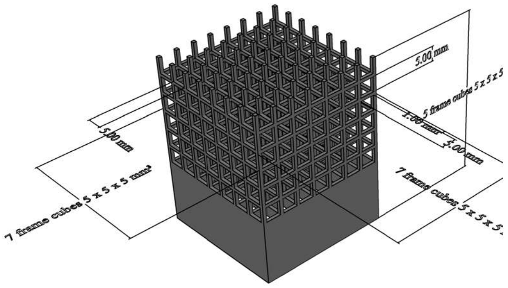

(2) Grid 5x5x1: rame cube 5x5x5 mm^3, wall thickness 1 mm

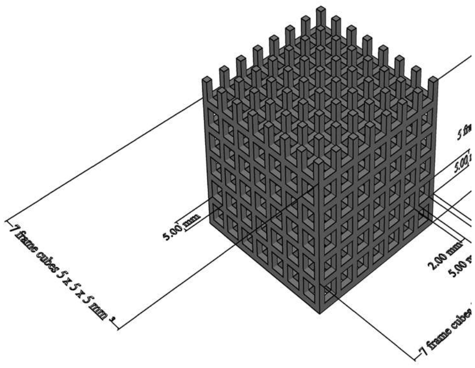

(3) Grid 5x5x2: frame cube 5x5x5 mm^3, wall thickness 2 mm

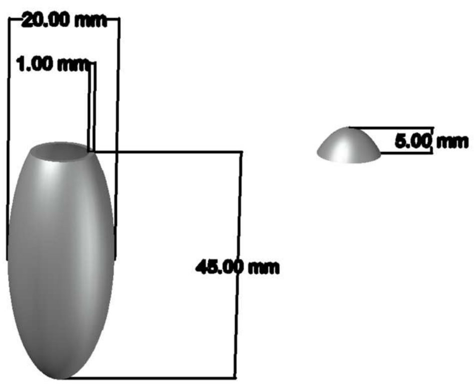

(4) Egg 5x2: Egg and its cap had 50 mm length, 20 mm diameter and 1 mm thickness

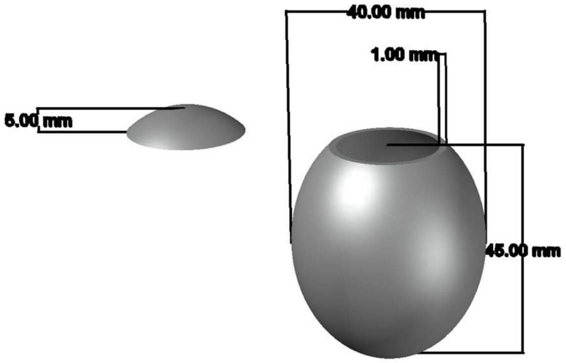

(5) Egg 5x4: Egg and its cap had 50 mm length, 40 mm diameter and 1 mm thickness

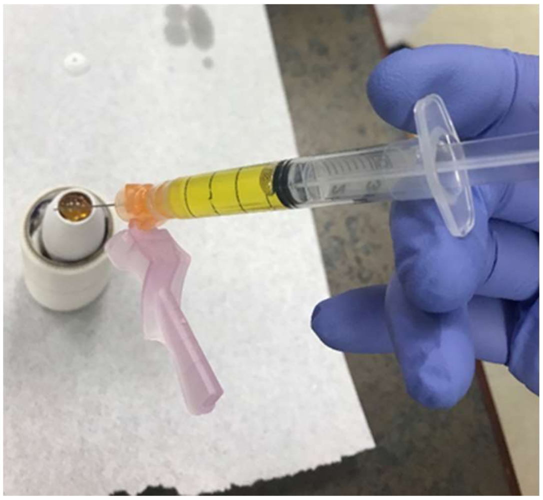

Vitamin D3 capsules were placed in in the egg-shaped objects, and voids filled up with olive oil.

Section B: Phantom setup procedure distributed to each institution

1. Setup the radiomics phantom

- Use distilled water to fill the radiomics phantom up to the water level marked on both lateral sides.
- Use the sagittal laser to place the radiomics phantom at the center of the imaging plane.
- Check the phantom direction along the head-foot (superior-inferior) direction.

2. MRI of the radiomics phantom

- Use an anterior and posterior array coil (i.e., an anterior chest or abdominal array coil, and a posterior array coil).
- Place the anterior coil just above the radiomics phantom (close enough).
- Scan the radiomics phantom 10 times. If possible, please re-setup the radiomics phantom every time.

Section C: Radiomic Feature Groups and Features

This following table names 107 radiomic features and their groups. We calculated 107 radiomic features, mentioned in Table 3.3 and parsed by feature group.

Feature Groups	Features
Shape	Elongation
	Flatness
	Least Axis Length
	Major Axis Length
	Maximum2DDiameterColumn
	Maximum2DDiameterRow
	Maximum2DDiameterSlice
	Maximum3DDiameter
	Mesh Volume
	Minor Axis Length
	Sphericity
	Surface Area
	Surface Volume Ratio
	Voxel Volume
First Order Statistic (First Order)	10Percentile
	90Percentile
	Energy
	Entropy
	Interquartile Range
	Kurtosis
	Maximum
	Mean Absolute Deviation
	Mean
	Median
	Minimum

	Range
	Robust Mean Absolute Deviation
	Root Mean Squared
	Skewness
	Total Energy
	Uniformity
	Variance
Gray Level Co-occurrence Matrix (glcm)	Autocorrelation
	Cluster Prominence
	Cluster Shade
	Cluster Tendency
	Contrast
	Correlation
	Difference Average
	Difference Entropy
	Difference Variance
	Inverse Difference (Id)
	Inverse Difference Moment (Idm)
	Inverse Difference Moment Normalized (Idmn)
	Inverse Difference Normalized (Idn)
	Information Measure of Correlation 1 (Imc1)
	Information Measure of Correlation 2 (Imc2)
	Inverse Variance
	Joint Average
	Joint Energy
	Joint Entropy
	Maximal Correlation Coefficient (MCC)
	Maximum Probability

	Sum Average
	Sum Entropy
	Sum Squares
Gray Level Dependence Matrix (gldm)	Dependence Entropy
	Dependence Non Uniformity
	Dependence Non Uniformity Normalized
	Dependence Variance
	Gray Level Non Uniformity
	Gray Level Variance
	High Gray Level Emphasis
	Large Dependence Emphasis
	Large Dependence High Gray Level Emphasis
	Large Dependence Low Gray Level Emphasis
	Low Gray Level Emphasis
	Small Dependence Emphasis
	Small Dependence High Gray Level Emphasis
	Small Dependence Low Gray Level Emphasis
Gray Level Run Length Matrix (glrlm)	Gray Level Non Uniformity
	Gray Level Non Uniformity Normalized
	Gray Level Variance
	High Gray Level Run Emphasis
	Long Run Emphasis
	Long Run High Gray Level Emphasis
	Long Run Low Gray Level Emphasis
	Low Gray Level Run Emphasis
	Run Entropy
	Run Length Non Uniformity
	Run Length Non Uniformity Normalized

	Run Percentage
	Run Variance
	Short Run Emphasis
	Short Run High Gray Level Emphasis
	Short Run Low Gray Level Emphasis
Gray Level Size Zone Matrix (glszm)	Gray Level Non Uniformity
	Gray Level Non Uniformity Normalized
	Gray Level Variance
	High Gray Level Zone Emphasis
	Large Area Emphasis
	Large Area High Gray Level Emphasis
	Large Area Low Gray Level Emphasis
	Low Gray Level Zone Emphasis
	Size Zone Non Uniformity
	Size Zone Non Uniformity Normalized
	Small Area Emphasis
	Small Area High Gray Level Emphasis
	Small Area Low Gray Level Emphasis
	Zone Entropy
	Zone Percentage
	Zone Variance
Neighboring Gray Tone Difference Matrix (ngtdm)	Busyness
	Coarseness
	Complexity
	Contrast
	Strength

Section D: Intraclass Correlation Coefficients

Intraclass correlation coefficients (ICC) are calculated for two ViewRays as described in the section 2.4.2.2.

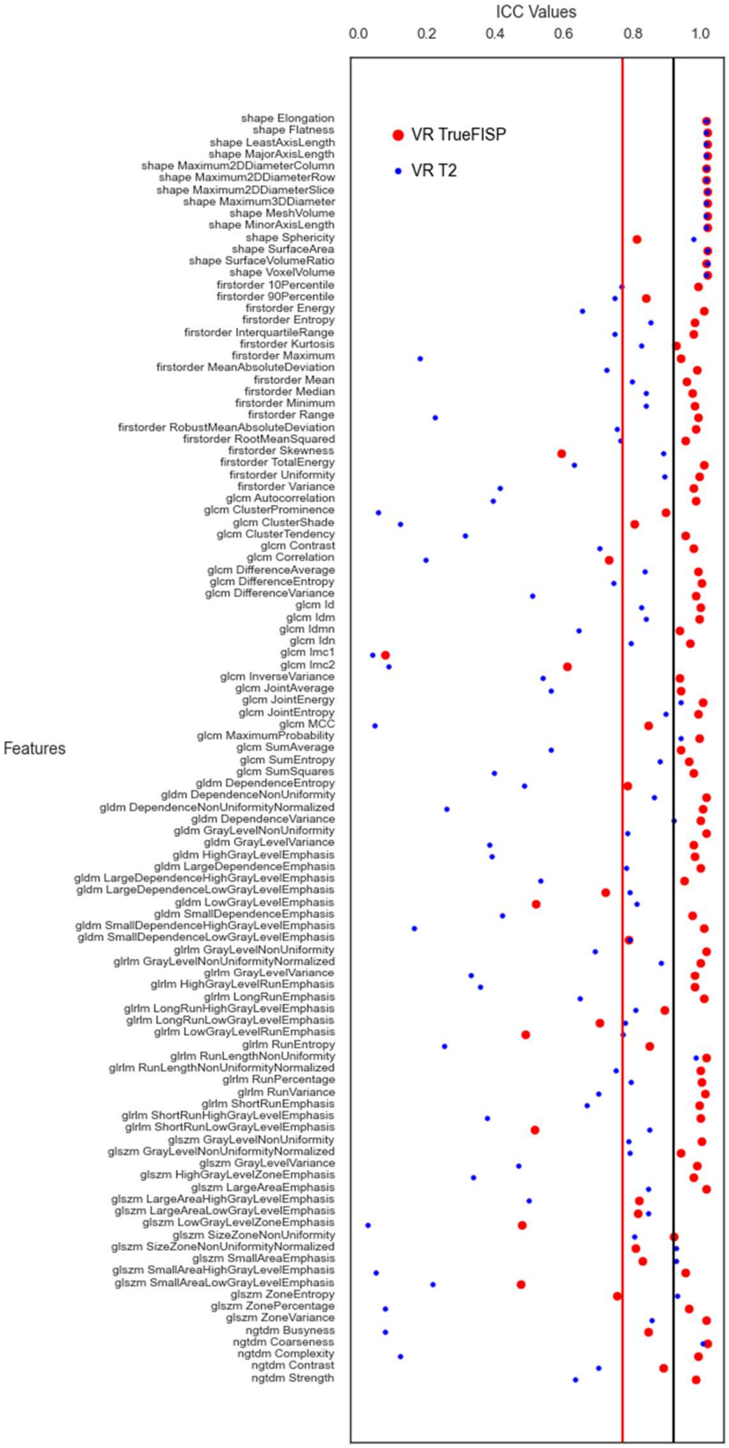

Supplement: Supplementary file 1 [file DataSheet1.pdf]
